# Income and Health in Tanzania. An Instrumental Variable Approach

**DOI:** 10.1016/j.worlddev.2014.09.016

**Published:** 2015-02

**Authors:** Eleonora Fichera, David Savage

**Affiliations:** a University of Manchester, UK; b HCL, UK

**Keywords:** Tanzania, rainfall shocks, income, health, spatial interpolation

## Abstract

There is a substantial debate over the direction of the causal relation between income and health. This is important for our understanding of the health production process and for the policy debate over improving healthcare. We instrument income with rainfall measurements by matching satellite information on timing and positioning of 21 rainfall stations to longitudinal data (1991–94) of over 4,000 individuals in 51 villages in Tanzania. A 10% increase in income reduces the number of illnesses by 0.02. We also find that a 10% increase in income implies an increase of about 0.1 vaccinations of children under six.

## 1 Introduction

There is a large literature that associates income and health. Despite the strength of this correlation, substantial debate continues over the direction of causation. Income affects health (Ettner, 1996, Smith, 1999) as individuals with more income live in healthier areas and can afford better healthcare. But health can also affect income, as healthier individuals are able to work more productively (Levy, 2000, McClellan, 1998, Wu, 2003). A final alternative is that both income and health are determined by other factors, such as time preferences (Barsky, Juster, Kimball, & Shapiro, 1997).

The nature of this relationship is important for our understanding of the health production and consumption process, but also for the public policy debate over improving healthcare (see for example, Attanasio & Hoynes, 2000).

Establishing a causal link between income and health requires an appropriate instrumental variables strategy. In the United Republic of Tanzania, the country we focus on in this paper, about 38% of the population lives below the national basic needs poverty line (National Bureau of Statistics, Tanzania National Budget Survey, 2007). Agriculture accounts for about half of gross production and employs about 80% of the labor force (Thurlow & Wobst, 2003). Agriculture in Tanzania is primarily rain-fed, with only 2% of arable land having irrigation infrastructure (FAO, 2009). Its main staple crops, like maize, are particularly susceptible to weather conditions. Because of this high dependence on weather events, we use meteorological data as an instrument for income.

Many studies in developing countries examine the effect on health of income shocks provoked by natural disasters (see for example, Bengtsson, 2010, Datar et al., 2013, Edoka, 2013, Miller and Urdinola, 2010, Pörtner, 2010, Rose, 1999, Yamano et al., 2005).

The principal contribution of this paper to the literature is a novel construction of rainfall data, using satellite measurements of historical rainfall data linked to individual-level longitudinal data. We use the timing and positioning of 21 weather stations across 51 villages from satellite data to generate a village-specific rainfall measurement through spatial interpolation of distances of the stations to the center of the village. We then match households’ interview dates, which vary by as much as seven months, to historical rainfall data and allow for spatial correlation in the covariance matrix.

The lack of variation in the instrument is a drawback of the previous literature. For instance, Rose (1999) linked household-level data, the Indian Additional Rural Incomes Survey, to district-level rainfall data collected by the World Bank. Bengtsson (2010), with the same Kagera data as in this paper, uses a time series of rainfall to instrument for income shocks and to identify causal impacts on body weight. However, he only exploits differences between rainfall measurements of five districts with only one weather measurement per district.

Using satellite-linked data to interview dates not only allows us to exploit within-villages and between-households variations, but also provides an “objective” measure of income shock. For instance, Edoka (2013) uses self-reported measures of weather shocks, which might be biased by measurement error, if exposure to weather shocks is correlated with differential perception of the impact on household’s welfare (Dercon, 2002).

An additional contribution of this paper is that we examine a wide range of health outcomes (i.e., Body Mass Index (BMI), number of self-reported illnesses; height-for-age, and weight-for-height for children under the age of six) and a preventive behavior, the number of vaccinations for children under the age of six. The World Health Organisation (WHO) considers these indicators as risk factors for other health problems and predictors of infant mortality. In our main specification, we find no statistically significant effect of transitory income changes on BMI, height-for-age, or weight-for-height. However, we do find a reduction in illnesses, such as acute fever, chills, coughs, severe headaches, and abdominal pain. In contrast, the main analysis of Bengtsson (2010) uses only one transitory health outcome measure, namely, relative weight defined as the deviation of individual weight from its mean. He finds a statistically significant increase in relative weight but only for females. We have been able to replicate his results with our novel construction of the instrument.

Only one study, by Miller and Urdinola (2010) does focus on the effect of income shocks on vaccinations and health outcomes for children. They examine the effect of world coffee price fluctuations on infant and child mortality in Colombia and find evidence of increased mortality and reduction of vaccinations for children under the age of one, which they attribute to an increase in the opportunity cost of time spent on childcare. By contrast, we find an increase in the number of vaccinations for children under the age of six. We are unable to measure the time spent on childcare directly, but we infer from the finding that rainfall variation in our data does not change the overall time spent on work, that it does not change. Compared with Miller and Urdinola (2010), the weather-related changes to income are smaller than coffee price variations and hence do not substantially change the opportunity cost of time. Like in Colombia, vaccination is almost entirely “free” in the Kagera region of Tanzania. But the distance to the nearest health center can often be quite large. We therefore infer that the prevailing income effect over the substitution effect in our data might be due to better access to healthcare centers (i.e., via transport mode).

The studies that found worse nutritional status in children have focused on small-scale natural disasters (see for example, Datar et al., 2013, Edoka, 2013, Pörtner, 2010) as opposed to the transitory weather changes we examine in this paper.

Our final contribution is the extensive discussion over the validity of our instrument, which is generally neglected by the literature. Weather shocks could affect health in two ways. Firstly, they could have a direct effect through morbidity and mortality. Secondly, they could impact the demand for health inputs, through their effect on income. There is empirical evidence suggesting the direct effect of weather shocks on health occurs when such shocks are extreme and not transitory like in our case (Deschêne and Greenstone, 2011, Burgess et al., 2011). Nevertheless, like Bengtsson (2010), we assess whether our rainfall measure has a direct effect on health with a specific focus on malaria and we find no such effect. Secondly, one novelty of our paper is the use of a number of additional instruments from the National Aeronautics and Space Administration (NASA)-linked climate data, such as temperature, humidity, and wind speed to indirectly test for the validity of rainfall. If rainfall could be argued to directly affect diseases such as malaria, this is less likely with wind speed. The test for the over-identifying restriction cannot reject the null hypothesis of uncorrelated residuals with the set of exogenous instruments. Our results are robust to checks around omitted variable bias, intra-household correlation, attrition rates and non-linear specification of our outcome models.

The paper is structured as follows. Section 2 contains a description of the data; summary statistics are reported in Section 3. The empirical strategy is explained in Section 4, while Section 5 reports the results and Section 6 concludes.

## 2 Data description

We link together three data sources, namely, the Kagera Health and Development Survey (KHDS), the Tanzania meteorological rainfall data, and the National Aeronautics and Space Administration (NASA) climate data.

### (a) Kagera health and development survey (KHDS)

We use baseline data from a longitudinal Living Standards Measurement Survey (LSMS) conducted in the Kagera region of North Western Tanzania,^1^ the Kagera Health and Development Survey (KHDS). It is one of the few long-running surveys containing questions on individual socioeconomic characteristics, wealth and income, health behaviors, and outcomes. KHDS also contains a rich set of community characteristics on health care, children’s education, and local market prices.

The Kagera region is predominantly rural and lies just south of the equator, bordering to the north with Uganda and to the west with Rwanda and Burundi. The population of 1.9 million people is predominantly engaged in agricultural production of banana and coffee in the north, and livestock and rain-fed annual crops, primarily cotton, maize, and sorghum, in the south. The agricultural sector accounts for 45% of GDP. About 29% of all households in Kagera are below the basic needs poverty line (Kessy, 2005). In 1991, household average annual expenditure was US$217 per capita, with a range of US$118 and US$357 across the six districts of the Kagera region.

**Figure 1:**
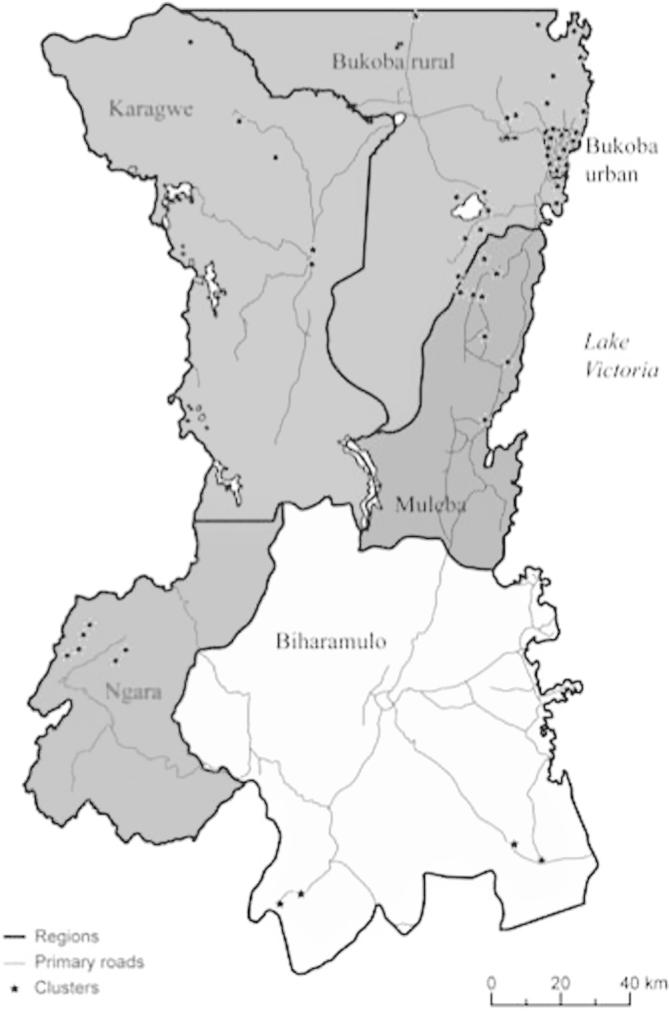
Map of the Kagera region with districts and sampled clusters.

Source: Luzi (2010)

The longitudinal survey consisted of four initial waves from 1991 to 1994.^2^ The first survey consisted of 915 households interviewed up to four times, from September 1991 to January 1994 (at 6–7-month intervals). Households were drawn from 51 villages (“clusters”) of 16 households each in the six administrative districts of Kagera: Biharamulo, Bukoba Rural, Bukoba Urban, Karagwe, Muleba, and Ngara. Figure 1 displays a map of the region showing the districts and the sampled villages.

The numbering of the “wave” is defined with respect to the timing of the interview across the whole sample, whereas “passage” is defined with reference to the number of interviews conducted with a specific household. Thus a replacement household, interviewed during the fourth wave would be its first passage, as it would the first time it had been interviewed. In the first passage, 840 households were interviewed. By the end of the fourth passage only 9.6% of the 840 had dropped out, which is a very low attrition rate for a developing country data set.

The random sample was stratified geographically according to mortality rates. A variable probability sampling selection procedure was used, based on predicted mortality: households with a similar predicted mortality replaced those that dropped out before the fourth wave.

### (b) Tanzania meteorological data

We link KHDS to monthly rainfall data collected by the Tanzania Meteorological Agency.^3^ Historical rainfall data are available for each month between January 1990 and December 1994. KHDS individual interview dates and the cluster where the individual is located are used to link to the meteorological data. Each household member interviewed on any day of a given month is assigned the average monthly rainfall measurement of the 12 months prior to the interview date.

The data contain the total millimeters (mm) of rain per month and the total days of rain per month for 21 weather stations in the Kagera region. It also reports the distance of the first and second nearest weather stations to the center of the cluster obtained from the Global Positioning System (GPS).^4^

While the Meteorological data is quite accurate, there are some values missing. The closest station to the center of the village has a proportion of non-missing data that range from a minimum of 78.2% to a maximum of 86.6% depending on the month of the year. The second-closest station to the center of the village has a proportion of non-missing data ranging from 62.5% to 82.1%. We replace missing information in either station by using the data from the other, which reaches between 88% and 94% of non-missing data. Remaining missing data have been replaced with the average rainfall measurement for that weather station.

### (c) NASA climate data

We also use the National Aeronautics and Space Administration (NASA) climate data linked to the 51 clusters using GPS coordinates.^5^

The data contain information on air temperature (in degrees Celsius, °C), relative humidity ratio (in percentage) at two meters above the surface of the Earth and wind speed at 10 m above the surface of the Earth (in meters per second). The relative humidity-ratio is the amount of actual water vapor in the air divided by the maximum amount of water vapor the air can hold at a given temperature. Relative humidity measures how close the air is to being saturated with water vapor. The wind gradient or wind speed gradient is the vertical gradient of the mean horizontal wind speed in the lower atmosphere. It measures the rate of increase of wind strength with unit increase in height above ground level.

Historical NASA climate data are available for every day and month during 1990–94. KHDS individual interview dates and the cluster where the individual is located are used to link to the climate data. Each household member is assigned the average climate measurements of the 12 months prior to their interview date.

## 3 Summary statistics

We describe each of the outcome variables of interest, the covariates and instrumental variables (Table 1). In order to examine the direction of the income–health relationship, we then correlate income with the outcome variables (Table 2).

**Table 1:** Definition of variables and descriptive statistics, KHDS (1991–94)

Variables	Variables definition	Obs.	Mean	S.D.	Min.	Max.
*Outcome variables:*						
Ln(BMI)	Natural logarithm of Body Mass Index	17,821	2.89	0.19	2.14	3.88
No. of conditions	Self-reported health symptoms in the past 4 weeks	17,847	0.90	1.07	0	5
Height-for-age	*z*-scores of height for age of children under the age of 6	3,215	−0.15	2.69	−8.76	5.83
Weight-for-height	*z*-scores of weight for height of children under the age of 6	3,212	−0.05	1.31	−14.06	9.15
No. of vaccinations	No. of vaccinations against measles, tetanus, TB, and polio of children under the age of 6	3,220	2.29	1.12	0	4

*Covariate variables:*						
Age	Age in years	17,847	22.31	20.10	0	110
Age squared	Squared value of age in years	17,847	901.92	1463.5	0	12100
Age*Female	Age in years of female respondent	17,847	12.18	18.61	0	110
Household size	No. of household members.	17,847	8.41	4.19	1	36
Ln(income)	Natural logarithm of real equivalized household income in TZS	17,847	10.77	1.11	0	14.78
Ln(education expenditure)	Natural logarithm of real equivalized household education expenditure in TZS	17,845	4.52	2.84	0	10.84
Ln(health expenditure)	Natural logarithm of real equivalized household health expenditure in TZS	17,852	5.11	2.14	0	12.11
Ln(savings)	Natural logarithm of real equivalized household cash savings in TZS	17,852	5.82	2.72	0	14.45
Distance to health center	Distance of nearest health center, clinic, hospital to the village in Kms	17,852	8.10	9.15	0	67

*Instrumental variables:*						
Ln(rainfall)	Natural logarithm of average monthly rainfall in previous 12 months (mm)	17,344	4.83	0.31	4.18	5.65
Ln(rainfall rainy season)	Natural logarithm of average monthly rainfall in rainy season during previous 12 months (mm)	17,344	5.31	0.34	4.49	6.12
Ln(rainfall dry season)	Natural logarithm of average monthly rainfall in dry season during previous 12 months (mm)	17,344	4.22	0.34	3.59	5.23
Ln(temperature)	Natural logarithm of average air temperature at 2 m above the surface of the earth (°C)	17,847	3.14	0.05	3.01	3.19

Note that statistics on covariates and instrumental variables are calculated on the sample of households reporting the no. of conditions.

**Table 2:** Outcome variables by income quintiles

Income quintiles	Bottom quintile	2nd quintile	3rd quintile	4th quintile	Top quintile
Ln(BMI)	2.88	2.89	2.89	2.89	2.91
	(3,580)	(3,550)	(3,568)	(3,574)	(3,545)
No. of conditions	0.96	0.93	0.86	0.86	0.87
	(3,586)	(3,561)	(3,576)	(3,576)	(3,548)
Height-for-age	−0.22	−0.25	−0.28	0.03	−0.05
	(628)	(650)	(656)	(678)	(603)
Weight-for-height	−0.25	−0.04	0.06	−0.02	−0.02
	(627)	(649)	(655)	(678)	(597)
No. of vaccinations	2.21	2.34	2.25	2.35	2.30
	(628)	(652)	(657)	(679)	(604)

*Note:* Sample means displayed and number of observations in ( ).

### (a) Description of the outcome variables

We focus on several measures of health used by the WHO: BMI, self-reported health conditions, height-for-age and weight-for-height *z*-scores, and number of vaccinations. BMI, height-for-age, and weight-for-height indicate malnutrition and are also considered risk factors for other health problems. The WHO considers these indicators in its Global Action Plan for the prevention and control of non-communicable diseases 2013–20.^6^ Self-reported conditions are considered a good proxy for the health status (Kroeger, 1983) and the number of vaccinations is an indicator of prevention against childhood mortality, one of the health-related Millennium Development Goals (MDGs). We will now examine the definition and construction of each of these variables in the Kagera data.

BMI is calculated as the ratio of weight (in kilograms) and the squared value of height (in meters). We take the natural logarithm of BMI. After taking out 35 observations with either very low or high BMI (less than 8 or more than 50), we are left with almost 18,000 observations across the four waves. It should be noted that less than 1% of the sample is obese (i.e., BMI greater than 30) and, on average, individuals in this sample are underweight (i.e., BMI lower than 18.5). Consequently, we interpret higher BMI as better health.

The household survey includes, for every individual, up to five self-reported health symptoms in the preceding four weeks: the most popular categories were acute fever, chills, coughs, severe headaches, and abdominal pain. 48% of the sample had no self-reported illnesses, 27% had one, 17% had two, and about 10% more than two. We construct a discrete, ordinal variable, which indicates the number of conditions from zero to five. Table 1 reports an average of about one condition for each individual.

Anthropometric measures for children under the age of six are height-for-age and weight-for-height. Height-for-age is a measure of stunting or chronic malnutrition and weight-for-height is a measure of wasting, acute or transitory malnutrition. The WHO suggests stunting to be a measure of social deprivation (WHO, 1986). Under the assumption that healthy children follow similar growth patterns across different populations, children‘s anthropometric measurements are standardized according to the International Referenced Population defined by the U.S. National Centre for Health Statistics (NCHS) with the Centers for Disease Control (CDC) and the World Health Organization (WHO, 1995). Therefore anthropometric measurements are expressed as *z*-scores: that is, a child‘s measurements and gender is compared to those of a similar child in a reference, healthy population defined by the U.S. NCHS, who has a *z*-score with mean zero and standard deviation of one. A severely stunted child has a *z*-score less than −3 and a *z*-score greater than −1 is not-stunted. On average there is no evidence of chronic or transitory malnutrition as the *z*-scores of height-for-age and weight-for-height in Table 1 are both greater than −1, but there are very large variations in the data which we will examine by income quintiles.

Health preventative behavior is proxied by the number of vaccinations against measles, tetanus, tuberculosis, and polio. We construct an ordinal variable, taking values from zero, if the child has no vaccinations, to four, if she has all four. Table 1 shows that on average children have been given approximately two vaccinations.

### (b) Description of the covariate variables

We consider a range of demographic variables such as household size, age in years, and its squared value. The sample of individuals is relatively young with an average age of about 22 years. Table 1 reports an average number of household members of about eight.

The principal income variable we use in the regressions is the natural logarithm of real income per capita, which is calculated at the household level for each wave. We define income as the sum of five components to account for possible spillovers of weather shocks: (i) employment income (i.e., income received as an employee of a private individual or of an institution other than the household for remuneration in cash or in kind); (ii) income from self-employment in agriculture (i.e., computed from gross revenues less costs of household-level activities in farming, livestock, and fishing, plus the value of home agricultural production); (iii) income from rent (e.g., income from renting land, farm equipment, dwellings, and rental value of owner-occupied housing); (iv) transfer income from individuals and organizations; and (v) other non-labor income (e.g., pension or retirement fund, insurance, interest on bank accounts, income from games, dowry, and inheritance). We omit non-farm self-employment, which represents 7% of all income, as the KDHS User Guide (World Bank, p.78) cautions that it is especially problematic, and in checking the individual data, only six out of over 3,000 data points have no income for all the other income categories.

To calculate per capita income, we use age- and gender-specific nutrition weightings, from a WHO reference scale (Dercon & Krishnan, 1998, p. 44), for each individual within the household. For each household, we then calculate an adult-equivalent size and divide the household income by this variable, relying on the assumption that households behave as a unitary model, with income distributed across household members based on nutritional requirements. Finally, we use the Fisher Ideal Index to deflate incomes. It corrects prices both temporally and spatially focusing on consumption baskets and budget shares of households in the Kagera region.^7^

The recall periods for questions concerning income were different across waves. In the first wave, the recall period was 12 months, whereas, for the other waves the preamble to the question was: “In the last six months or since I was last here” (World Bank, 2004, p. 90). We therefore annualise the data from waves two to four by doubling it. Individuals in our sample have an average income of about US$200 per year at the 2004 exchange rate.

The health of individuals might also be affected by household expenditure on education, health, and cash savings in the previous 12 months. Education expenditure refers to items such as school contributions, clothes, books, transport, and other items. Health expenditure includes medicines, other medical services, transport, and hospitalization. On average households spend less than 1% of their income on such items.

The distance individuals need to travel to the closest health center; clinic or hospital may also affect their health. We consider the distance of the village as opposed to the distance of each individual household because the latter measure contains over 50% of missing values. On average a health center is about 8 km from the village but there are large variations across villages.

Table 2 has descriptive statistics of the outcome variables by income quintiles. As income increases the number of self-reported illness symptoms decrease slightly from an average of 0.96 to an average of 0.87. Number of vaccinations increases particularly between the bottom and second quintile. Comparing the bottom and top quintiles of income, it seems that children become more nourished and BMI increases slightly.

### (c) Description of the instrumental variables

The Kagera region has been affected by severe droughts in the late 1980s, outside the period of interest to this paper. We exploit the timing and quantity of rainfall and timing of temperature as exogenous shocks to income and analyze their effect on health outcomes and behaviors over the period of interest. We consider three dimensions of our instrumental variables, namely, their cross-sectional variation, relevance, and validity.

The cross-sectional variation in our instruments for each interview passage has two sources: timing and geography. In order to adequately reflect the crop cycle, we construct a continuous variable, which represents for each region the average monthly rainfall of the 12 months prior to the month of interview in the KHDS. We also account for seasonal variation taking the average rainfall in the rainy (March–May, October–November) and in the dry (June–September, December–February) seasons during the 12 months preceding the month of interview in the KHDS.

Timing variation occurs because the interview date, within a passage, can vary by as much as seven months. Geographical variation is generated because there are 21 weather stations and each of the 51 clusters is assigned measurements of the two closest stations. In order to determine a monthly rainfall measurement that varies between clusters, we use the distances and rainfall measurements of the two closest stations to the center of the village. Spatial interpolation has been performed using Inverse Distance Weighting squared (IDW) as follows:

R^vt=∑i=1NwiRit

$$w_{i}=\frac{d_{i}^{-2}}{\sum_{i=1}^{N}d_{i}^{-2}}$$

where R^vt indicates the unknown rainfall measurement in millimeters (mm) at the center of *v* = 1,…, 51 villages, in time *t* = 1,…, 12 (months); *R_it_* is the rainfall measurement (mm) of each of the *N* = 2 closest weather stations *i*.

The intuition of IDW is that the interpolating surface is a weighted average of the location of the weather stations and the weight assigned to each station diminishes as the distance from the center of the village increases. This interpolation technique has been shown to be accurate in determining the actual rainfall measurement of an unknown point (see for example, Chen and Liu, 2012, Dirks et al., 1998, Zhuang and Wang, 2003).

Time variation in temperature derives from interview dates. We construct a variable that accounts for the average temperature faced by each household member in the 12 months prior to their interview date. Geographical variation comes from the fact that daily temperature measurements are provided for each of the 51 villages.

The relevance of the instruments is supported by the high dependence of the population on agricultural production making them vulnerable to both the timing and the quantity of rainfall and the timing and level of temperature. This issue is examined in more detail in sub-section 5(a).

Rainfall is a valid instrument if it affects health only through income and is not correlated with the residuals of our outcome regressions. There is empirical evidence that the direct effect of weather shocks on health occurs when such shocks are extreme and not transitory, as in our case (Burgess et al., 2011, Deschêne and Greenstone, 2011). For instance Burgess *et al*. (2011) found that an additional day with mean temperature over 36 Celsius degrees (°C) relative to a day with temperature around 22–24 °C in India increases annual mortality rate by 0.75%. It could be argued that, for example, rainfall increases the incidence of malaria directly, via an increased population of mosquitos around stagnant water. There are two objections that we can make against this argument. Firstly, we might argue that the relation between rainfall and diseases (and even mortality in the mentioned studies) goes through income, which is what we instrument for. In other words, even if the population of mosquitoes is higher in the rainy season, the incidence of malaria has a socioeconomic gradient. Secondly, we can use a number of additional instruments, such as temperature, humidity, and wind speed to indirectly test for the validity of rainfall. If rainfall could be argued to affect health directly through diseases such as malaria, this is less likely with wind speed. This analysis will be examined in more detail in sub-section 5(b).

From Table 1 we can infer that the average monthly rainfall is about 125 mm, ranging between a minimum of 65 mm to a maximum of 284 mm. The difference between dry and rainy season is also sizeable with over 200 mm rain on average in the rainy season and 68 mm in the dry season. The average temperature is about 23.1 °C, ranging from a minimum of 20 °C to a maximum of 24 °C. The low variation in temperature can partly be attributed to its measurement being at the village level rather than weighted by the distance to the weather station, as in the case of rainfall data.

## 4 Empirical strategy

Our central results use two-stage least square regressions (2SLS):

(1) $$H_{i(j)t}=\beta {\mathrm{lnincome}}_{i(j)t}+\gamma X_{i(j)t}+\delta D_{\mathrm{jt}}+\eta_{i(j)}+\varepsilon_{\mathrm{ii}(j)}\;t=1\text{,}2\text{,}\ldots .\text{,}T\text{.}$$

where subscript *i*(*j*) denotes the individual living in village *j* and *t* time. *lnincome_i_*_(_*_j_*_)_*_t_* is the independent variable of interest, the logarithm of real income per capita, *β* the coefficient of interest, *X_i_*_(_*_j_*_)_*_t_* is a vector of demographic characteristics including age, its squared value and interaction with gender, household size, and, in some specifications, several wealth characteristics (expenditure and savings), *η_i_*_(_*_j_*_)_ is the unobserved time-invariant individual effects (i.e., the individuals’ fixed effects), and *ɛ_i_*_(_*_j_*_)_*_t_* the error term. Some specifications also include *D_jt_* indicating the distance of the village *j* to the closest health center. *H_i_*_(_*_j_*_)_*_t_* is the dependent variable, which may be any of the health outcomes or preventative health behavior. It is a continuous variable for BMI, weight-for height and height-for age and a count variable for the number of conditions and vaccinations.

There are several reasons why income might be endogenous. First, there is reverse causality as bad health might affect income. In the case of adult members, bad health might restrict their supply of labor, which, in turn, also affects health. Children’s poor health might also affect family income, if additional time has to be spent on childcare. Second, there may be omitted factors that affect both income and health. Such factors only matter if they are time-invariant, otherwise they would be captured by the individuals’ fixed effects.^8^ For instance, an individual’s discount factor could be an unobserved time-varying factor that affects both income and health. Third, there is measurement error of income itself. To assert that $\overset{ˆ}{\beta }$ is an estimate of the causal effect of income on health outcomes or behaviors, we use meteorological measurements for the period *t* − 1 as the instrumental variable *z*_*i*(*j*)*t*-1_ in the first-stage equation:

(2) $${\mathrm{lnincome}}_{i(j)t}=\alpha z_{i(j)t-1}+\theta_{i(j)}+v_{i(j)t}$$

all models include a set of cluster and year interaction variables and a linear time trend.^9^

In the main models we use robust standard errors, using clustering at the “cluster” level (there are 51 clusters or villages in the sample), to account for heteroskedasticity.

Following Miguel, Satyanath, and Sergenti (2004), and Wooldridge (2002), we use a 2SLS linear probability model for the discrete variables, as with our continuous dependent variables, rather than an ordered model, such as logit or probit, and do not account for the categorical dependent variables to be ordered.

For each of the 2SLS regressions, we adopt a similar reporting format. A health variable is the dependent variable, representing either health outcomes or behavior. The natural logarithm of real income per capita is the independent variable of interest. For the instrumental variable, we use the monthly average rainfall measurement (mm) in the 12 months prior to the interview date. We consider a 12-month period to comprise all stages of the crop cycle. But we also examine alternative definitions of rainfall including average monthly rainfall in the rainy and dry seasons during the 12 months prior to the interview.

In the robustness checks, we consider additional instruments from the NASA climate data such as temperature, humidity, and wind speed.

There are two ways in which weather shocks may affect health outcomes through income. Firstly, there are changes in income available to the individual for food consumption (the income effect). Secondly, changes in income affect the opportunity cost of time in health-promoting activities (the substitution effect). For instance, at times of favorable weather shocks, the income effect might work toward better health outcomes. The substitution effect works in the opposite direction and may result in health deterioration, if less time can be devoted to health improvement.

The case of vaccination is one that warrants specific attention. As the community data show that immunization is free for the majority of villages in the Kagera region, the income effect might not determine the ability to pay for the service, but the ability to access services (for instance, via transportation mode). During favorable weather shocks, the substitution effect works in the same direction as above. Which effect predominates in the case of health outcomes and behaviors of individuals facing income shocks is an empirical question.

## 5 Results

### (a) Main specifications

Table 3 reports a simple, linear, fixed effects model of the relation between income and health outcomes and behaviors. The OLS estimates suggest a positive and statistically significant correlation between income and health, indicating an increase in BMI and weight-for-height, a reduction in number of self-reported illness symptoms, and an increased uptake of vaccinations. A 10% increase in income is associated with an increase of 0.05 in the take up of vaccinations. For a median individual, with a BMI value of 17.7, a 10% increase in income is associated with an increase in BMI of about 0.01. However, reverse causality, omitted variable bias, or measurement error in the income/health relation may bias the results and this prevents us from making any causal inference.

**Table 3:** Basic OLS models

	Model I: Ln(BMI)	Model II: No. conditions	Model III: Height-for-age	Model IV: Weight-for-height	Model V: No. vaccinations
Ln(income)	0.005⁎⁎⁎	−0.06⁎⁎⁎	−0.04	0.16⁎⁎⁎	0.46⁎⁎⁎
	(0.001)	(0.02)	(0.06)	(0.04)	(0.09)
Age	0.003⁎	0.01	–	–	0.38⁎⁎⁎
	(0.002)	(0.03)			(0.09)
Age squared	−0.00003⁎⁎⁎	0.0001	–	–	−0.07⁎⁎⁎
	(0.00)	(0.0002)			(0.01)
Age⁎Female	0.002	0.03⁎⁎	–	–	−0.02
	(0.001)	(0.02)			(0.05)
HH size	−0.001	0.02	0.05	−0.02	−0.03
	(0.001)	(0.01)	(0.04)	(0.03)	(0.03)
Constant	2.90⁎⁎⁎	0.76	0.23	−1.62⁎⁎⁎	−2.48⁎⁎
	(0.03)	(0.55)	(0.78)	(0.50)	(1.03)
No. of observations	*17,821*	*17,847*	*3,215*	*3,212*	*3,220*
No. of individuals	*6,130*	*6,136*	*1,322*	*1,321*	*1,324*

*Note:* Std. errors in () clustered by villages. All models include year-cluster interaction effects, a month linear time trend, and individual fixed effects.

⁎⁎⁎ *p* < 0.01.

⁎⁎ *p* < 0.05.

⁎ *p* < 0.10.

Table 4, Table 5 display the results of the 2SLS models, instrumented with rainfall. We choose these as our main models because rainfall data allow us to exploit a more refined geographical variation at the cluster level than the NASA data. First-stage regressions (i.e., Eqn. 2) suggest a positive and statistically significant association between rainfall and income. A 1% increase in average monthly rainfall increases income by approximately 2.8 percentage points. Kleibergen and Paap (2006) provide a Wald *F*-statistic for the null hypothesis of weak identification of the instrument. The critical value of 16.38 suggested by Baum, Schaffer, and Stillman (2007) implies a rejection of the null.

**Table 4:** 2SLS models of health outcomes

	Model I		Model II	
	1st stage: Ln(Income)	2nd stage: Ln(BMI)	1st stage: Ln(Income)	2nd stage: No. conditions
Ln(rainfall)	2.83⁎⁎⁎	–	2.84⁎⁎⁎	–
	(0.36)		(0.35)	
Ln(income)	–	0.01⁎	–	−0.15⁎⁎⁎
		(0.003)		(0.05)
Age	−0.02	−0.002	0.02	0.01
	(0.02)	(0.002)	(0.02)	(0.03)
Age squared	0.0002	−0.00004⁎⁎	−0.0002	0.00001
	(0.0002)	(0.00)	(0.0002)	(0.0002)
Age*Female	0.02⁎	0.002⁎	0.02⁎	0.03⁎⁎
	(0.01)	(0.001)	(0.01)	(0.02)
HH size	0.02	−0.001	0.02	0.01
	(0.02)	(0.001)	(0.02)	(0.01)
No. of Observations	*16,083*		*16,105*	
No. of individuals	*4,845*		*4,848*	
Kleibergen–p *F*-stat (*H*_0_ = weak IV)	*63.72*		*64.05*	

*Note:* Std. errors in () clustered by villages. All models include year-cluster interaction effects, a month linear time trend, and individual fixed effects.

⁎⁎⁎ *p* < 0.01.

⁎⁎ *p* < 0.05.

⁎ *p* < 0.10.

**Table 5:** 2SLS models of health outcomes and behaviors

	Model I		Model II		Model III	
	1st stage: Ln(Income)	2nd stage: Height-for-age	1st stage: Ln(Income)	2nd stage: Weight-for-age	1st stage: Ln(Income)	2nd stage: No. vaccinations
Ln(rainfall)	2.59⁎⁎⁎	–	2.58⁎⁎⁎	–	2.58⁎⁎⁎	–
	(0.44)		(0.44)		(0.44)	
Ln(income)	–	0.02	–	0.12	–	1.00⁎⁎⁎
		(0.13)		(0.17)		(0.16)
Age	–	–	–	–	0.14⁎	0.29⁎⁎⁎
					(0.08)	(0.09)
Age squared	–	–	–	–	−0.02⁎⁎⁎	−0.06⁎⁎⁎
					(0.01)	(0.01)
Age*Female	–	–	–	–	0.03	−0.02
					(0.04)	(0.06)
HH size	0.02	0.07	0.02	−0.03	0.02	−0.04
	(0.02)	(0.04)	(0.02)	(0.03)	(0.02)	(0.04)
No. of Obs.	*2,696*		*2,694*		*2,699*	
No. of individuals	*899*		*898*		*899*	
Kleibergen–Paap *F*-stat (*H*_0_ = weak IV)	*34.49*		*34.39*		*34.68*	

*Note:* Std. errors in () clustered by villages. All models include year-cluster interaction effects, a month linear time trend, and individual fixed effects. ^∗∗^*p* < 0.05.

⁎⁎⁎ *p* < 0.01.

⁎ *p* < 0.10.

While we find a weakly statistically significant and positive effect of income on BMI, we do observe a significant reduction in the number of self-reported illness symptoms. A 10% increase in income decreases the number of illnesses by 0.02.

In Table 5 we do not report any significant effect of income on height-for-age. This can be explained by the fact that height-for-age is a typical measure of chronic malnutrition and the one-period time difference between any two waves coupled with the transitory nature of rainfall shocks might not be enough to capture the long-term effects on health. A larger effect can be noticed on the uptake of preventative health measures. A 10% increase in income increases the number of vaccinations of children under the age of six by approximately 0.1.

The main results do not change even when considering the average rainfall in the rainy and dry seasons as reported in Table 6, Table 7.

**Table 6:** 2SLS models of health outcomes with seasonal instruments

	Model I		Model II	
	1st stage: Ln(Income)	2nd stage: Ln(BMI)	1st stage: Ln(Income)	2nd stage: No. conditions
Ln(rainfall in rainy season)	1.44⁎⁎⁎	–	1.44⁎⁎⁎	–
	(0.35)		(0.35)	
Ln(rainfall in dry season)	1.28⁎⁎⁎		1.28⁎⁎⁎	
	(0.27)		(0.27)	
Ln(income)	–	0.01	–	−0.15⁎⁎⁎
		(0.004)		(0.05)
Age	−0.02	−0.002	−0.02	0.01
	(0.02)	(0.002)	(0.02)	(0.03)
Age squared	0.0002	−0.00004⁎⁎	0.0002	0.0001
	(0.0002)	(0.00002)	(0.0002)	(0.0002)
Age*Female	0.02⁎	0.002⁎	0.02⁎	0.03⁎⁎
	(0.01)	(0.001)	(0.01)	(0.02)
HH size	0.02	−0.001	0.02	0.01
	(0.02)	(0.001)	(0.02)	(0.01)
No. of Observations	*16,083*		*16,105*	
No. of individuals	*4,845*		*4,848*	
Kleibergen–Paap *F*-stat (*H*_0_ = weak IV)	*36.34*		*36.54*	

*Note:* Std. errors in () clustered by villages. All models include year-cluster interaction effects, a month linear time trend, and individual fixed effects.

⁎⁎⁎ *p* < 0.01.

⁎⁎ *p* < 0.05.

⁎ *p* < 0.10.

**Table 7:** 2SLS models of health outcomes and behaviors with seasonal instruments

	Model I		Model II		Model III	
	1st stage: Ln(Income)	2nd stage: Height-for-age	1st stage: Ln(Income)	2nd stage: Weight-for-age	1st stage: Ln(Income)	2nd stage: No. vaccinations
Ln(rainfall in rainy season)	1.55⁎⁎⁎	–	1.55⁎⁎⁎	–	1.56⁎⁎⁎	–
	(0.40)		(0.40)		(0.40)	
Ln(rainfall in dry season)	0.99⁎⁎⁎		0.99⁎⁎⁎		0.98⁎⁎⁎	
	(0.31)		(0.31)		(0.31)	
Ln(income)	–	−0.03	–	0.10	–	0.99⁎⁎⁎
		(0.12)		(0.17)		(0.16)
Age	–	–	–	–	0.13⁎	0.29⁎⁎⁎
					(0.07)	(0.09)
Age squared	–	–	–	–	−0.02⁎⁎	−0.06⁎⁎⁎
					(0.01)	(0.01)
Age*Female	–	–	–	–	0.03	−0.02
					(0.04)	(0.06)
HH size	0.02	0.07	0.02	−0.02	0.02	−0.04
	(0.02)	(0.04)	(0.02)	(0.03)	(0.02)	(0.04)
No. of Obs.	*2,696*		*2,694*		*2,699*	
No. of individuals	*899*		*898*		*899*	
Kleibergen–Paap *F*-stat (*H*_0_ = weak IV)	*18.58*		*18.53*		*18.69*	

*Note:* Std. errors in () clustered by villages. All models include year-cluster interaction effects, a month linear time trend, and individual fixed effects.

⁎⁎⁎ *p* < 0.01.

⁎⁎ *p* < 0.05.

⁎ *p* < 0.10.

The positive and significant effect on the number of vaccinations might indicate that the income effect prevails over the substitution effect in these data. We have attempted to identify possible mechanisms through which an increase in vaccinations might have occurred. Unlike in Miller and Urdinola (2010), we do not have data on time spent on childcare, but we found that weather shocks do not significantly affect overall working hours (defined as the amount of time in a week spent on working the land, cooking, or collecting wood).^10^ We might therefore infer that the time spent on childcare has not changed either. Given the variation in distances to the health center, income might have affected transportation access to such facilities.

Differences in the statistical significance and size of different health outcomes could be explained by the transitory nature of income shocks. Rainfall shocks (other than droughts as in the case of our data) create a temporary shift in household income. Table 4, Table 5 show that transitory income shocks do not have statistically significant effects on long-term measures of health outcomes such as BMI or height-for-age. This is in contrast to Bengtsson (2010) transitory measure of relative body weight. But there is evidence of a reduction in the number of conditions such as cough, fever, diarrhea, and an increase in the vaccinations to children under the age of six. The latter might have long-term effects in terms of life expectancy, but more data would be needed to determine whether such is the case for this population.

### (b) Robustness checks

In this sub-section we examine the robustness of our results to issues around omitted variables, instrument construction, its validity, intra-household correlation effects, attrition rates, and non-linear specification of some of our models.

In Table 8, Table 9, Table 10 we address concerns regarding the omission of potentially important determinants such as education and health expenditure, savings, and distance to a health center. Our results are robust to the inclusion of these additional controls, but we share the concerns expressed by Dell, Jones, and Olken (2014) regarding the “over-controlling” problem in a weather regression, where we cannot be sure that these additional controls are indeed exogenous. The similarity in the size and significance of our main results alleviates such concerns.

It might be argued that using the logarithm of the average monthly rainfall in the 12 months prior to the interview reduces the variability in the instrument. We therefore use the average monthly rainfall without taking its natural logarithm. After using these alternative instruments in separate models, we find our main results to be unchanged.^10^

One argument against the validity of our instrument could be that rainfall affects health directly through an increase in the incidence of diseases such as malaria. Instrument validity cannot be directly tested, but we offer two arguments against this objection.

Firstly, we test whether rainfall affects malaria directly with a linear probability fixed effects model of the effect of rainfall on malaria. In the KHDS, individuals are asked whether a medical practitioner has diagnosed them with malaria. In Table 11 of Appendix A we show that rainfall has no statistically significant effect on malaria, at least in this sample. This result confirms that of Bengtsson (2010) on the same data and it holds even using temperature as an alternative instrument (see Model II in Table 11).

Secondly, we use a number of additional instruments, such as temperature, humidity, and wind speed from the NASA climate data to indirectly test for the validity of rainfall. In Table 12, Table 13, we show that temperature is also significantly and negatively correlated with income. For instance, a 1% increase in temperature decreases income by about 26%.^11^ The size of this effect is comparable to the literature (see for example Dell et al., 2009, Dell et al., 2014, Di Falco et al., 2012). For a selection of eleven countries in South America and for U.S., Dell *et al*. (2009) found that a 1 °C increase in temperature is associated with a 1.2% decline in labor income (very close to a 1.5% decline in our measure of income when temperature is not in logarithm format^10^). With two instruments we can test for over-identifying restrictions using the Hansen *J*-test. Under the null hypothesis the residuals should be uncorrelated with the set of exogenous variables if the instruments are truly exogenous. We cannot reject the null for any of the outcome models. This result also holds with the other instruments such as humidity and wind speed.^10^ Wind speed in particular, might be argued less likely to affect health directly.

Table 14, Table 15 report results of the same models of health outcomes and behavior, where the standard errors have been clustered by household. If households behave as a unitary model, food consumption and health behaviors are likely to be correlated between household members. There is no difference in the size or statistical significance of the models in Table 14, Table 15 compared to Table 4, Table 5. However, income has a statistically significant effect at the 5% level on BMI.

Attrition is always a concern of longitudinal household surveys, especially where health is of interest, as death or illness can be a significant determinant of non-response. As the primary objective of the KHDS survey was to “estimate the economic impact of the death of prime-age adults on surviving household members” (World Bank, 2004, p. 6), it was particularly important that attrition rates be minimized: for example, if household dissolution and migration are significant coping strategies, following an adult death, then attrition might introduce material bias into the sample. By the end of the fourth passage, 9.6% of the 840 households interviewed in the first passage had dropped out (World Bank, 2004, p. 6).

To check for the robustness of the results to attrition bias, we re-run a sample of the regressions, using a balanced panel of households that completed all four waves, to compare whether the estimates produced are similar. In Table 16, Table 17 we show the sign and size of the effect of income on health to be generally unchanged.

As a final robustness check, we use a non-linear, correlated, random-effects ordered probit model for the number of illnesses and vaccinations. We use a two-stage residual inclusion (2SRI) method, which has been shown to be consistent for two-stage instrumental variable non-linear models (for a proof see Terza, Basu, and Rathouz (2008)). The first stage consists of a linear regression of income on rainfall. Second-stage regressions are ordered probit models of number of illnesses and vaccinations against income and the first-stage residual. Intuitively, conditional on the first-stage residuals, income is considered to be exogenous in the second-stage regression. The results are exactly the same as in the linear model with a larger effect for lower income.^9^^,^^10^

## 6 Conclusions

In this paper, we examined whether income shocks affect a range of health outcomes and preventative behaviors by matching satellite information on timing and positioning of 21 rainfall stations to individual-longitudinal data in 51 villages of the Kagera region in Tanzania. Agriculture accounts for about half of gross production and employs about 80% of the labor force in Tanzania, making rainfall measurements an ideal instrument for income shocks.

We find a positive effect of income on health. A 10% increase in income reduces the number of illnesses by 0.02. A further finding is the positive effect on vaccinations of children under six: a 10% increase in income implies an increase of approximately 0.1 vaccinations, from a mean of 2.3 per child, for the four vaccinations of polio, tetanus, tuberculosis, and measles. We have attempted to examine some of the mechanisms through which the vaccination rate might rise as a result of an increase in income. As there is no evidence that rainfall shocks affect the number of working hours and vaccinations are mostly free, we infer that the positive relation might be attributed to lower transportation costs or time to reach the health center. Unfortunately we do not have household-level data to ascertain whether this is the correct mechanism, but we can see in our data that some health centers are very far from the village.

Our results are robust to the inclusion of additional controls, to different definitions of rainfall and to using other climate data as instrumental variables, to non-linear specifications of our model, potential attrition biases, and intra-household correlation in health outcomes and behaviors.

The policy implication of these results for developing countries is that increasing households’ income is likely to have significant secondary benefits in terms of improving both health outcomes and behaviors. We have discussed that one potential channel could be improved access to healthcare centers.

## Figures and Tables

**Table 8:** Basic OLS Models (with additional controls)

	Model I: Ln(BMI)	Model II: No. conditions	Model III: Height-for-age	Model IV: Weight-for-height	Model V: No. vaccinations
Ln(income)	0.006⁎⁎⁎	−0.07⁎⁎⁎	−0.03	0.16⁎⁎⁎	0.45⁎⁎⁎
	(0.001)	(0.02)	(0.06)	(0.04)	(0.09)
Age	−0.003	0.01	–	–	0.38⁎⁎⁎
	(0.002)	(0.03)			(0.09)
Age squared	−0.00003⁎	0.0001	–	–	−0.07⁎⁎⁎
	(0.00002)	(0.0003)			(0.01)
Age*Female	0.002	0.03⁎	–	–	−0.02
	(0.001)	(0.02)			(0.05)
HH size	−0.001	0.01	0.05	−0.02	−0.03
	(0.001)	(0.03)	(0.04)	(0.03)	(0.03)
Ln(education expenditure)	−0.0001	−0.002	0.02	−0.005	0.02
	(0.001)	(0.01)	(0.02)	(0.02)	(0.02)
Ln(health expenditure)	−0.0003	0.06⁎⁎⁎	−0.002	0.01	0.004
	(0.0004)	(0.01)	(0.02)	(0.01)	(0.01)
Ln(savings)	−0.0002	−0.005	0.02	−0.01	−0.001
	(0.0004)	(0.01)	(0.02)	(0.01)	(0.01)
Distance to health center	−0.0003	0.003	0.01	−0.01	−0.02
	(0.0004)	(0.01)	(0.01)	(0.01)	(0.01)
Constant	2.90⁎⁎⁎	0.63	0.01	−1.56⁎⁎⁎	−2.34⁎⁎
	(0.03)	(0.60)	(0.80)	(0.55)	(1.00)
No. of observations	*17,814*	*17,840*	*3,214*	*3,211*	*3,219*
No. of individuals	*6,130*	*6,136*	*1,322*	*1,321*	*1,324*

*Note:* Std. errors in () clustered by villages. All models include year-cluster interaction effects, a month linear time trend and individual fixed effects.

⁎⁎⁎ *p* < 0.01.

⁎⁎ *p* < 0.10.

⁎ *p* < 0.10.

**Table 9:** 2SLS models of health outcomes (with additional controls)

	Model I		Model II	
	1st stage: Ln(Income)	2nd stage: Ln(BMI)	1st stage: Ln(Income)	2nd stage: No. conditions
Ln(rainfall)	2.79⁎⁎⁎	–	2.79⁎⁎⁎	–
	(0.34)		(0.34)	
Ln(income)	–	0.01⁎	–	−0.17⁎⁎⁎
		(0.004)		(0.05)
Age	−0.02	−0.002	−0.02	0.01
	(0.02)	(0.002)	(0.02)	(0.03)
Age squared	0.0002	−0.0004⁎⁎	0.0002	0.0001
	(0.0002)	(0.00002)	(0.0002)	(0.0003)
Age*Female	0.02⁎	0.002⁎	0.02⁎	0.03⁎⁎
	(0.01)	(0.001)	(0.01)	(0.02)
HH size	0.01	−0.001	0.01	0.01
	(0.02)	(0.001)	(0.02)	(0.01)
Ln(education expenditure)	0.02⁎	−0.0001	0.02	−0.003
	(0.01)	(0.0005)	(0.01)	(0.01)
Ln(health expenditure)	0.03⁎⁎⁎	−0.0002	0.03⁎⁎⁎	0.06⁎⁎⁎
	(0.01)	(0.0004)	(0.01)	(0.01)
Ln(savings)	0.01⁎	−0.0002	0.01⁎	−0.003
	(0.01)	(0.0004)	(0.01)	(0.01)
Distance to health center	−0.001	−0.0002	−0.001	0.002
	(0.02)	(0.0002)	(0.02)	(0.005)
No. of Observations	*16,075*		*16,097*	
No. of individuals	*4,844*		*4,847*	
Kleibergen–Paap *F*-stat (*H*_0_ = weak IV)	*66.97*		*67.33*	

*Note:* Std. errors in () clustered by villages. All models include year-cluster interaction effects, a month linear time trend, and individual fixed effects.

⁎⁎⁎ *p* < 0.01.

⁎⁎ *p* < 0.05.

⁎ *p* < 0.10.

**Table 10:** 2SLS models of health outcomes and behaviors (with additional controls)

	Model I		Model II		Model III	
	1st stage: Ln(Income)	2nd stage: Height-for-age	1st stage: Ln(Income)	2nd stage: Weight-for-age	1st stage: Ln(Income)	2nd stage: No. vaccinations
Ln(rainfall)	2.50⁎⁎⁎	–	2.49⁎⁎⁎	–	2.49⁎⁎⁎	–
	(0.41)		(0.41)		(0.41)	
Ln(income)	–	0.05	–	0.11	–	1.01⁎⁎⁎
		(0.14)		(0.17)		(0.16)
Age	–	–	–	–	0.13⁎	0.29⁎⁎
					(0.07)	(0.09)
Age squared	–	–	–	–	−0.02⁎⁎⁎	−0.06⁎⁎⁎
					(0.01)	(0.01)
Age*Female	–	–	–	–	0.04	−0.02
					(0.04)	(0.06)
HH size	0.02	0.07⁎	0.02	−0.02	0.01	−0.04
	(0.02)	(0.04)	(0.02)	(0.03)	(0.02)	(0.04)
Ln(education expenditure)	0.03	0.01	0.03	−0.003	0.03	0.004
	(0.02)	(0.02)	(0.02)	(0.02)	(0.02)	(0.02)
Ln(health expenditure)	0.04⁎⁎⁎	−0.003	0.04⁎⁎⁎	0.02	0.04⁎⁎⁎	−0.02
	(0.01)	(0.03)	(0.01)	(0.01)	(0.01)	(0.02)
Ln(savings)	0.02⁎	−0.03	0.02⁎	−0.01	0.02	−0.01
	(0.01)	(0.02)	(0.01)	(0.01)	(0.01)	(0.01)
Distance to health center	0.002	0.02⁎	0.002	−0.01	0.002	−0.01
	(0.02)	(0.01)	(0.02)	(0.01)	(0.02)	(0.01)
No. of Obs.	*2,695*		*2,693*		*2,698*	
No. of individuals	*899*		*898*		*899*	
Kleibergen–Paap *F*-stat (*H*_0_ = weak IV)	*36.97*		*36.58*		*31.45*	

*Note:* Std. errors in () clustered by villages. All models include year-cluster interaction effects, a month linear time trend, and individual fixed effects.

⁎⁎⁎ *p* < 0.01.

⁎⁎ *p* < 0.05.

⁎ *p* < 0.10.

**Table 11:** Model of health outcome (Malaria)

	Model I	Model II
Ln(rainfall)	−0.05	–
	(0.41)	
Ln(temperature)	–	5.40
		(7.26)
Age	0.02	0.004
	(0.06)	(0.06)
Age squared	0.0001	0.0002
	(0.0005)	(0.0004)
Age*Female	−0.03	−0.01
	(0.04)	(0.04)
HH size	0.05⁎⁎	0.04
	(0.02)	(0.02)
Constant	−0.05	−17.09
	(2.38)	(22.76)
*No.ofObservations*	*1,767*	*1,811*
*No.ofindividuals*	*1,437*	*1,465*

*Note:* Std. errors in () clustered by villages. All models include year-cluster interaction effects, a month linear time trend, and individual fixed effects. ^∗∗∗^*p* < 0.01. ^∗^*p* < 0.01.

⁎⁎ *p* < 0.05.

**Table 12:** 2SLS outcome models (with additional instrument)

	Model I		Model II	
	1st stage: Ln(Income)	2nd stage: Ln(BMI)	1st stage: Ln(Income)	2nd stage: No. conditions
Ln(rainfall)	2.43⁎⁎⁎	–	2.43⁎⁎⁎	–
	(0.41)		(0.41)	
Ln(temperature)	−25.66⁎⁎	–	−25.67⁎⁎	
	(11.75)		(11.73)	
Ln(income)	–	0.01⁎	–	−0.13⁎⁎
		(0.004)		(0.06)
Age	−0.02	−0.002	−0.02	0.01
	(0.02)	(0.002)	(0.02)	(0.03)
Age squared	0.0002	−0.00004⁎⁎	0.0002	0.0001
	(0.0002)	(0.00002)	(0.0002)	(0.0002)
Age*Female	0.02⁎⁎	0.002⁎	0.02⁎⁎	0.03⁎⁎
	(0.01)	(0.001)	(0.01)	(0.02)
HH size	0.01	−0.001	0.01	0.01
	(0.02)	(0.001)	(0.02)	(0.01)
No. of Observations	*16,083*		*16,105*	
No. of individuals	*4,845*		*4,848*	
Hansen *J* over-identification test	*0.86* *p-value* = *0.35*		*1.13* *p-value* = *0.29*	

*Note:* Std. errors in () clustered by villages. All models include year-cluster interaction effects, a month linear time trend, and individual fixed effects.

⁎⁎⁎ p < 0.01.

⁎⁎ p < 0.05.

⁎ p < 0.10.

**Table 13:** 2SLS models of outcomes and behaviors (with additional instrument)

	Model I		Model II		Model III	
	1st stage: Ln(Income)	2nd stage: Height-for-age	1st stage: Ln(Income)	2nd stage: Weight-for-age	1st stage: Ln(Income)	2nd stage: No. vaccinations
Ln(rainfall)	2.20⁎⁎⁎	–	2.20⁎⁎⁎	–	2.19⁎⁎⁎	–
	(0.52)		(0.46)		(0.51)	
Ln(temperature)	−24.99⁎⁎	–	−25.17⁎⁎	–	−25.05⁎⁎	
	(13.29)		(11.55)		(11.42)	
Ln(income)	–	0.02	–	0.12	–	0.96⁎⁎⁎
		(0.12)		(0.16)		(0.15)
Age	–	–	–	–	0.13⁎	0.29⁎⁎⁎
					(0.07)	(0.09)
Age squared	–	–	–	–	−0.02⁎⁎⁎	−0.06⁎⁎⁎
					(0.01)	(0.01)
Age*Female	–	–	–	–	0.04	−0.02
					(0.04)	(0.06)
HH size	0.01	0.07	0.01	−0.02	0.01	−0.04
	(0.02)	(0.04)	(0.02)	(0.03)	(0.02)	(0.04)
No. of Obs.	*2,696*		*2,694*		*2,699*	
No. of individuals	*899*		*898*		*899*	
Hansen *J* over-identification test)	*0.010* *p-value* =* 0.92*		*0.01* *p-value* =* 0.93*		*0.99* *p-value* =* 0.32*	

*Note:* Std. errors in () clustered by villages. All models include year-cluster interaction effects, a month linear time trend, and individual fixed effects.

⁎⁎⁎ *p* < 0.01.

⁎⁎ *p* < 0.05.

⁎ *p* < 0.10.

**Table 14:** 2SLS models of health outcomes (HH clustered std. errors)

	Model I		Model II	
	1st stage: Ln(Income)	2nd stage: Ln(BMI)	1st stage: Ln(Income)	2nd stage: No. conditions
Ln(rainfall)	2.83⁎⁎⁎	–	2.84⁎⁎⁎	–
	(0.19)		(0.19)	
Ln(income)	–	0.01⁎⁎	–	−0.15⁎⁎⁎
		(0.003)		(0.04)
Age	−0.02	−0.002	−0.02	0.01
	(0.02)	(0.002)	(0.02)	(0.03)
Age squared	0.0002	−0.00004⁎⁎⁎	0.0002	0.0001
	(0.0001)	(0.00001)	(0.0001)	(0.0002)
Age*Female	0.02⁎	0.002⁎	0.02⁎	0.03⁎
	(0.01)	(0.001)	(0.01)	(0.02)
HH size	0.02	−0.001	0.02	0.01
	(0.02)	(0.001)	(0.02)	(0.01)
No. of Observations	*16,083*		*16,105*	
No. of households	*871*		*871*	
Kleibergen–Paap *F*-stat (*H*_0_ = weak IV)	*223.29*		*224.44*	

*Note:* Std. errors in () clustered by households. All models include year-cluster interaction effects, a month linear time trend, and individual fixed effects.

⁎⁎⁎ *p* < 0.01.

⁎⁎ *p* < 0.05.

⁎ *p* < 0.10.

**Table 15:** 2SLS models of health outcomes and behaviors (HH clustered std. errors)

	Model I		Model II		Model III	
	1st stage: Ln(Income)	2nd stage: Height-for-age	1st stage: Ln(Income)	2nd stage: Weight-for-age	1st stage: Ln(Income)	2nd stage: No. vaccinations
Ln(rainfall)	2.59⁎⁎⁎	–	2.58⁎⁎⁎	–	2.58⁎⁎⁎	–
	(0.20)		(0.20)		(0.20)	
Ln(income)	–	0.02	–	0.12	–	1.00⁎⁎⁎
		(0.16)		(0.12)		(0.13)
Age	–	–	–	–	0.14⁎⁎	0.29⁎⁎⁎
					(0.06)	(0.09)
Age squared	–	–	–	–	−0.02⁎⁎⁎	−0.06⁎⁎⁎
					(0.01)	(0.01)
Age*Female	–	–	–	–	0.03	0.02
					(0.04)	(0.06)
HH size	0.02	0.07	0.02	−0.03	0.02	−0.04
	(0.02)	(0.04)	(0.02)	(0.03)	(0.02)	(0.04)
No. of Obs.	*2,696*		*2,694*		*2,699*	
No. of households	*485*		*484*		*485*	
Kleibergen–Paap *F*-stat (*H*_0_ = weak IV)	*169.40*		*168.95*		*168.34*	

*Note:* Std. errors in () clustered by households. All models include year-cluster interaction effects, a month linear time trend, and individual fixed effects. ^∗^*p* < 0.10.

⁎⁎⁎ *p* < 0.01.

⁎⁎ *p* < 0.05.

**Table 16:** 2SLS models of health outcomes (balanced sample)

	Model I		Model II	
	1st stage: Ln(Income)	2nd stage: Ln(BMI)	1st stage: Ln(Income)	2nd stage: No. conditions
Ln(rainfall)	2.65⁎⁎⁎	–	2.65⁎⁎⁎	–
	(0.44)		(0.44)	
Ln(income)	–	0.01	–	−0.17⁎⁎
		(0.004)		(0.07)
Age	−0.02	0.0002	−0.02	0.03
	(0.02)	(0.002)	(0.02)	(0.03)
Age squared	0.0001	−0.0004⁎⁎	0.0001	0.0001
	(0.0002)	(0.00002)	(0.0002)	(0.0003)
Age*Female	0.02	0.003⁎	0.02	0.02
	(0.01)	(0.001)	(0.01)	(0.02)
HH size	0.03	−0.001⁎	0.03	0.01
	(0.02)	(0.001)	(0.02)	(0.01)
No. of Observations	*11,095*		*11,105*	
No. of individuals	*2,869*		*2,870*	
Kleibergen–Paap *F*-stat (*H*_0_ = weak IV)	*36.81*		*37.00*	

*Note:* Std. errors in () clustered by villages. All models include year-cluster interaction effects, a month linear time trend, and individual fixed effects.

⁎⁎⁎ *p* < 0.01.

⁎⁎ *p* < 0.05.

⁎ *p* < 0.10.

**Table 17:** 2SLS models of health outcomes and behaviors (balanced sample)

	Model I		Model II		Model III	
	1st stage: Ln(Income)	2nd stage: Height-for-age	1st stage: Ln(Income)	2nd stage: Weight-for-age	1st stage: Ln(Income)	2nd stage: No. vaccinations
Ln(rainfall)	2.52⁎⁎⁎	–	2.52⁎⁎⁎	–	2.52⁎⁎⁎	–
	(0.52)		(0.52)		(0.52)	
Ln(income)	–	0.12	–	0.20	–	1.10⁎⁎⁎
		(0.09)		(0.23)		(0.12)
Age	–	–	–	–	0.07	0.28⁎⁎
					(0.09)	(0.12)
Age squared	–	–	–	–	−0.01	−0.06⁎⁎⁎
					(0.01)	(0.02)
Age*Female	–	–	–	–	−0.02	0.03
					(0.04)	(0.07)
HH size	0.04	0.11⁎⁎	0.04	−0.05	0.04	−0.11⁎⁎⁎
	(0.03)	(0.05)	(0.03)	(0.03)	(0.03)	(0.04)
No. of Obs.	*1,691*		*1,691*		*1,693*	
No. of individuals	*491*		*491*		*491*	
Kleibergen–Paap *F*-stat (*H*_0_ = weak IV)	*23.52*		*23.52*		*23.33*	

*Note:* Std. errors in () clustered by villages. All models include year-cluster interaction effects, a month linear time trend, and individual fixed effects. ^∗^*p* < 0.10.

⁎⁎⁎ *p* < 0.01.

⁎⁎ *p* < 0.05.

## Footnotes

1 The data are publicly available on the World Bank website: http://econ.worldbank.org/WBSITE/EXTERNAL/EXTDEC/EXTRESEARCH/EXTLSMS/0,,contentMDK:21991269~menuPK:4196952∼pagePK:64168445∼piPK:64168309∼theSitePK:3358997,00.html.

2 KHDS 1991–94 was funded by the World Bank and Muhimbili University College of Health Sciences. Consult: http://econ.worldbank.org/WBSITE/EXTERNAL/EXTDEC/EXTRESEARCH/EXTLSMS/0,,contentMDK:21991269~menuPK:4196952∼pagePK:64168445∼piPK:64168309∼theSitePK:3358997,00.html for a detailed description of the original data and follow-up surveys.

3 Data are available from the Economic Development Initiatives website at http://www.edi-africa.com/research/khds/introduction.htm. Last accessed on 30th May 2013.

4 GPS is a space-based satellite navigation system that provides location and time information on or near the Earth. It was initially developed by the US Department of Defense in the early 1970s.

5 These data were obtained from the NASA Langley Research Center POWER Project funded through the NASA Earth Science Directorate Applied Science Program. It is freely available at: http://power.larc.nasa.gov/cgi-bin/cgiwrap/solar/hirestimeser.cgi?email=daily@larc.nasa.gov.

6 The report is available at: http://www.who.int/nmh/events/ncd_action_plan/en/.

7 It is provided in the Kagera data and its construction is explained at http://siteresources.worldbank.org/INTLSMS/Resources/3358986-1181743055198/3877319-1353959490637/khds2010_price_index_note.pdf.

8 We do not include education for three reasons. First, it contains a large portion of missing values. Second, education does not vary over time in developing countries, especially considering the short time-frame of our analysis. Finally, education is highly correlated with the income variable we have constructed. We do however, check the robustness of our results to the inclusion of expenditure and distance to the health center.

9 They have been estimated using stata v. 13 provided by StataCorp, Texas (USA).

10 These results are available from the authors on request.

11 In order to test whether the effect is due to the correlation between rainfall and temperature, we have run the same regression using temperature as the only instrument and the size of the effect is very similar. These results are available from the authors on request.
